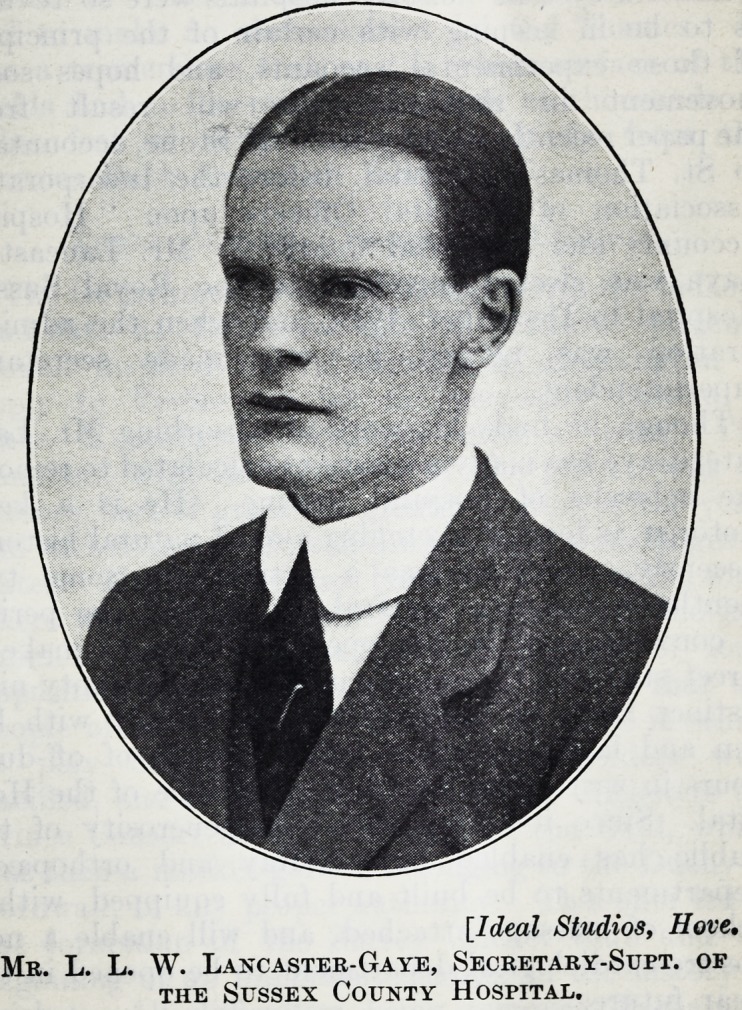# Hospital Men of Mark: Brigadier-General Brownlow and Mr. Lancaster-Gaye

**Published:** 1924-12

**Authors:** 


### HOSPITAL MEN OF MARK.

### BRIGADIER-GENERAL BROWNLOW AND MR. LANCASTER-GAYE.

It was in 1813 that the Earl of Egremont, Lord-Lieutenant of Sussex, called a meeting at the Old Ship, Brighton, to discuss the possibility of organising some medical charity on a broader basis than the one already in operation which was known as The Sussex General Infirmary. There followed the inevitable delay, and it was not till 1826 that Lord Egremont laid the foundation-stone of " The Sussex County Hospital and Sea-Bathing Infirmary." In

*Mendoza Galleries.*]
BRIGADIER-GENERAL D'ARCY BROWNLOW, C.M.G., C.I.E., CHAIRMAN OF THE SUSSEX COUNTY HOSPITAL.

those early days much importance attached to the latter portion of its title, and thousands of bathing tickets had to be issued. The bringing into the hospital of a daily supply of 250 gallons of sea-water was no small item of expense. In the first place it was maintained by conveying the water to the baths direct from the sea in a butt and dray, but subsequently it was obtained from the New Steine Hotel. Two of the many administrative duties of the first house surgeon were the supervision of a brewery, with the purchase of best pale malt and Kent or Sussex hops, and the control of the machinery of the well, including the provender issued to the engine horse which, apparently, supplied the power for raising the water only intermittently. In November, 1828, the Board of Management passed the following resolution : " That the following limit in respect of beer be enforced in the hospital, and that the matron be directed to see the same carried into effect : Nurses, 2 pints ; porters and washerwoman, 2 pints each ; the cook, housemaid and scullery maid, 1½ pints each per day."

The Royal Sussex County Hospital has always had for its patron the reigning monarch, and in its very early days it was " minutely inspected " by William IV. and Queen Adelaide. Four years ago Princess Mary opened the extended Nurses' Home, which is a beautiful building standing in a peculiarly advantageous position. The hospital possesses an excellent laboratory, said to surpass any laboratory in the kingdom unattached to an active medical

[*Ideal Studios, Hove.*
MR. L. L. W. LANCASTER-GAYE, SECRETARY-SUPT. OF THE SUSSEX COUNTY HOSPITAL.

school. A serious situation had to be faced in 1922 when the Committee were compelled to close 110 beds, but the crisis was overcome, and since September of last year the full services of the institution have been available. The hospital has as its chairman a distinguished soldier, who was for two years Principal Military Governor of Basra, Brigadier-General d'A. C. Brownlow, C.M.G., C.I.E. General Brownlow was elected to the Board of Management in 1922, and in view of his considerable administrative experience his election is a happy one. He succeeded Mr. Ball Dodson, who, although he has retired from the chair after twenty-nine years' connection with the Royal Sussex Hospital, still takes a keen interest in its welfare.

Mr. Lancaster-Gaye, the secretary-superintendent, is the son of the Rector of Skeyton, Norfolk, and was educated at St. Anne's Societies' School, Redhill, and Sir William Paston's Grammar School. Possessed of artistic tastes he first took up architectural work, and his spare time was spent in visiting and sketching ecclesiastical buildings in Norfolk, where he made a particular study of ancient sundials, consecration crosses and masons' marks. He was appointed to the secretarial staff of the Royal Sussex County Hospital in April, 1911, was elected assistant-secretary in October, 1912, and secretary-in-charge in July, 1914, in which office he remained until called up for military service in 1917. Owing to his experience of civil hospital accounting Mr. Lancaster-Gaye was posted to the R.A.M.C. and served as Unit accountant in various military hospitals, in one of which he initiated a complete system of cost accounts. He is strongly of opinion that civil hospitals would do well to take a lesson in many directions from the system entered upon by the War Office, and thinks that there could be more effective control over expenditure if the "uniform system" of civil hospitals were so revised as to be in keeping with certain of the principles of those experimental accounts, and hopes some movement in that direction will result from the paper recently read by Mr. J. E. Stone, accountant to St. Thomas's Hospital, before the Incorporated Association of Hospital Officers upon "Hospital Accounts and Financial Control." Mr. Lancaster-Gaye was elected secretary to the Royal Sussex Hospital in December, 1919, and when the administration was revised he was made secretary-superintendent.

Though he finds his work all-absorbing Mr. Lancater-Gaye has many recreations calculated to remove the cobwebs of hospital routine. He is a keen motorist, is fond of sketching and of natural history. Recently, while he was a patient for some two months in his own hospital, he utilised the period of convalescence on the open balconies to make a direct study of the songs and habits of twenty-nine distinct kinds of birds. He is also ready with his pen and last year spent many months of off-duty hours in writing an interesting Epitome of the Hospital. Since its publication the generosity of the public has enabled new X-Ray and orthopædic departments to be built and fully equipped, with a school of massage attached, and will enable a new electro-cardiological department to be opened in the near future.

## Figures and Tables

**Figure f1:**
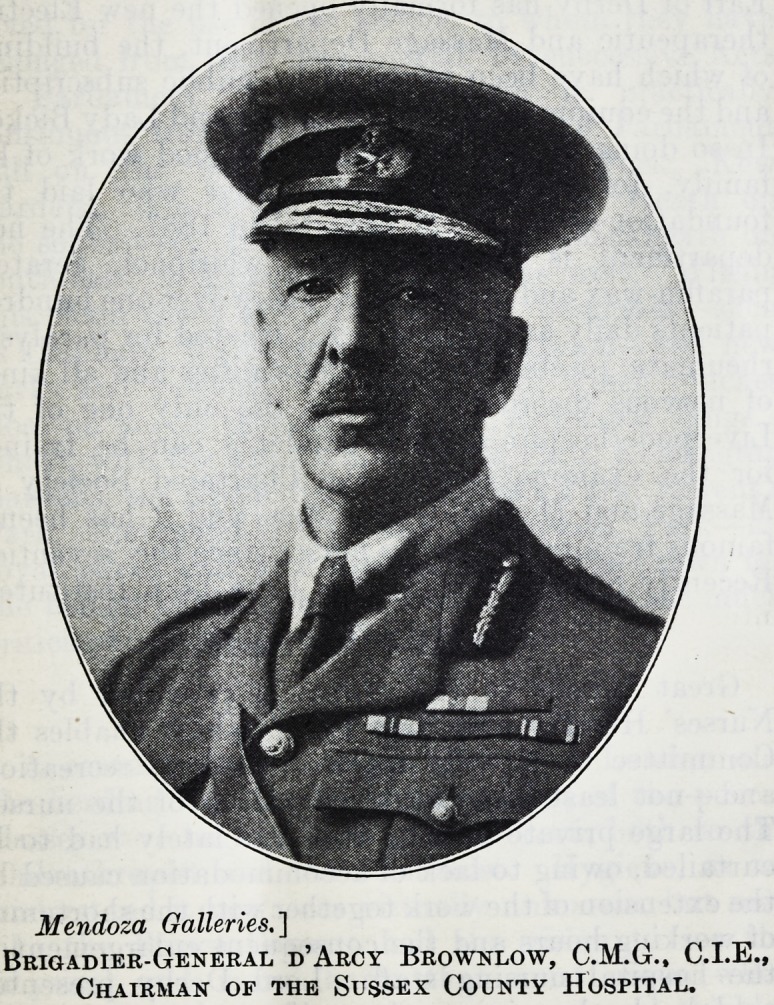


**Figure f2:**